# Retraction: PKCε activation restores loss of PKCε, manganese superoxide dismutase, vascular endothelial growth factor, and microvessels in aged and Alzheimer's disease hippocampus

**DOI:** 10.3389/fnagi.2026.1792359

**Published:** 2026-01-28

**Authors:** Frontiers Editorial Office

The journal retracts the March 2022 article cited above.

Following publication, concerns were raised regarding the integrity of the images in the published figures. Image duplication concerns were identified in Figure 8B panel AD II–III and Figure 8B panel AD IV–VI.

The authors have remained unresponsive to multiple requests for the raw data for this study. Given extant concerns over the validity of the study, the article has been retracted.

This retraction was approved by the Chief Editors of Frontiers in Aging Neuroscience and the Chief Executive Editor of Frontiers. The authors have not responded to correspondence regarding this retraction.

